# Non-Invasive Photoacoustic Imaging of *In Vivo* Mice with Erythrocyte Derived Optical Nanoparticles to Detect CAD/MI

**DOI:** 10.1038/s41598-020-62868-1

**Published:** 2020-04-06

**Authors:** Yonggang Liu, Taylor Hanley, Hao Chen, Steven R. Long, Sanjiv S. Gambhir, Zhen Cheng, Joseph C. Wu, Georges El Fakhri, Bahman Anvari, Raiyan T. Zaman

**Affiliations:** 1 Department of Medicine, Division of Cardiology, Stanford University School of Medicine, Stanford, CA USA; 2 Department of Bioengineering, University of California, Riverside, CA USA; 3 Department of Radiology, Stanford University School of Medicine, Stanford, CA USA; 4 Department of Pathology, University of California, San Francisco, CA United States; 5 Molecular Imaging Program at Stanford, Stanford University School of Medicine, Stanford, CA USA; 6 Department of Bioengineering, Stanford University School of Medicine, Stanford, CA USA; 7 Stanford Cardiovascular Institute, Stanford University School of Medicine, Stanford, CA USA; 8 Department of Radiology, Harvard Medical School, Boston, MA USA; 9 Gordon Center for Medical Imaging, Massachusetts General Hospital, Boston, MA USA

**Keywords:** Cardiovascular diseases, Interventional cardiology

## Abstract

Coronary artery disease (CAD) causes mortality and morbidity worldwide. We used near-infrared erythrocyte-derived transducers (NETs), a contrast agent, in combination with a photoacoustic imaging system to identify the locations of atherosclerotic lesions and occlusion due to myocardial-infarction (MI). NETs (≈90 nm diameter) were fabricated from hemoglobin-depleted mice erythrocyte-ghosts and doped with Indocyanine Green (ICG). Ten weeks old male C57BL/6 mice (n = 9) underwent left anterior descending (LAD) coronary artery ligation to mimic vulnerable atherosclerotic plaques and their rupture leading to MI. 150 µL of NETs (20 µM ICG,) was IV injected via tail vein 1-hour prior to photoacoustic (PA) and fluorescence *in vivo* imaging by exciting NETs at 800 nm and 650 nm, respectively. These results were verified with histochemical analysis. We observed ≈256-fold higher PA signal from the accumulated NETs in the coronary artery above the ligation. Fluorescence signals were detected in LAD coronary, thymus, and liver. Similar signals were observed when the chest was cut open. Atherosclerotic lesions exhibited inflammatory cells. Liver demonstrated normal portal tract, with no parenchymal necrosis, inflammation, fibrosis, or other pathologic changes, suggesting biocompatibility of NETs. Non-invasively detecting atherosclerotic plaques and stenosis using NETs may lay a groundwork for future clinical detection and improving CAD risk assessment.

## Introduction

Coronary artery disease (CAD) is the leading causes of death worldwide. The asymptomatic nature of CAD makes the early detection very difficult as not all CAD show up in a CT^1^, and MRI’s^2^ inability to accurately estimate plaque burden and their degree of stenosis make it difficult to predict heart attacks. Early clinical diagnosis of CAD is critical not only to prevent catastrophic events of plaque rupture through intervention or pharmacological strategy, but also contributes to the study of epidemiology of vulnerable plaques. Early detection of CAD non-invasively especially thin cap fibro atheroma (TCFA) or inflamed plaques in coronary artery is still difficult today due to its small size, motion, and obscuring signal from adjacent myocardium. Thus, catheterization x-ray angiography, an invasive method, is the clinical gold standard for CAD diagnosis in advance stage^3^. One of the major limitation of conventional coronary catheterization x-ray arteriography is how plaque is depicted as a silhouette rather than detecting positively remodeled lesions, specifically TCFAs, which do not impinge on the lumen significantly^4^.

The information on plaques pathobiology and their targets by non-invasive imaging is important. Thus, in nuclear imaging, the level of uptake of the most widely used PET tracer fluorodeoxygluocose (FDG) is directly correlated with the number of plaque macrophage infiltration in most inflamed arteries compared to non-diseased arteries^5^. Aortic inflammation specifically giant-cell arteritis and Takayasu arteritis in patients with large-vessel vasculitis were detected and quantified through ^11^C labeled tracer-PK11195 in conjunction of PET/CT^6^. Iron oxide accumulation by macrophages, the hallmarks of vulnerable atherosclerotic composite plaque inflammation, can be imaged with MRI in human^7,8^. Iron-oxide nanoparticles has traditionally been imaged with T2*-weighted MRI gradient echo sequences^9^. These sequences create robust contrasts in parenchymal organs such as the myocardium (heart), liver, and pancreas by generating signal hypo-intensity or negative contrast surrounding the area of iron-oxide nanoparticles. However, it is difficult to image vessel walls with this approach due to lack of suitable background for negative contrast from the presence of large amounts of air in the structures surrounding the vessel wall in thorax and neck areas. Recently, photoacoustic (PA) imaging is being used for detecting plaque-specific lipid accumulation in collagen structure and thrombosis of carotid artery between 1130–1250 nm wavelength^10,11^. However, no studies have ever targeted inflammatory cells as a molecular marker for plaque vulnerability with PA imaging.

We are presenting here a novel near infrared (NIR) contrast agent called NIR erythrocyte-derived transducers (NETs) in conjunction with a PA and fluorescence imaging systems to detect inflammation in coronary artery. We hypothesized that during PA imaging, when a non-ionizing laser pulse is delivered to the coronary artery, it will able to detect stenosis or blockage due to thermoelastic expansion from energy absorption by NETs within inflammatory cells. These expansions will be detected as acoustic pressure wave by the ultrasound transducer and generate PA signal. Herein we demonstrate for the first time that NETs-mediated PA imaging provides a capability for non-invasive detection of inflammation as well as the location of advanced stage of stenosis and occlusion in the left anterior descending (LAD) coronary artery ligation mice models.

## Materials and Methods

### Preparing mice LAD coronary ligation model

All methods were performed in accordance with the guidelines and regulations approved by the Administrative Panel on Laboratory Animal Care at the Stanford University School of Medicine (APLAC #18086). The use of LAD coronary artery ligation mice model in this study is two-fold: (1) find the location of inflammation caused my macrophages accumulation due to injury from the ligation surgery; and (2) identify the occlusion/stenosis of coronary artery or location of MI. Ten weeks old male C57BL/6 (Jax Lab) mice (n = 9) from Jackson Laboratory were used for LAD coronary artery ligation procedure. After 7 days of acclamation based on an approved protocol (APLAC #18086), the LAD ligation surgical procedure was performed. Mice were anesthetized with isoflurane before subjected to the ligation surgery. The mice were intubated and opened the chest cavity at the left intercostal space to expose the heart and the LAD artery. Once the LAD artery was distinguished under the dissecting microscope, an 8–0 polypropylene suture was placed under the LAD artery for permanent ligation with the suture. After the whole procedure, the chest was closed using 6-0 polypropylene suture. Mice were then extubated and monitored until fully awake and active. These mice were healed for one week before the imaging experiment.

### NETs fabrication

NETs were fabricated from Swiss Webster Sentinel mice red blood cells. Blood was drawn from these mice using syringes coated with 3% heparin followed by erythrocytes separation from the whole blood via centrifugation (1000 × g for 5–10 minutes at 4 °C). The plasma and buffy coat were removed before the erythrocyte pellet was re-suspended in ≈ 320 mOsm phosphate buffered saline (PBS) (defined as 1 × PBS), centrifuged (1000 × g for 5–10 minutes at 4 °C) and washed with 1 × PBS three times. The supernatant was discarded after each wash. The erythrocytes were then subjected to hypotonic treatment by re-suspension in 0.25 × PBS (≈80 mOsm) and were allowed to incubate for ≈10 minutes before being centrifuged (20,000 × g for 20 minutes at 4 °C). The supernatant containing hemoglobin was discarded, and the hypotonic treatment was repeated until the erythrocyte pellet was white in color, indicating the presence of hemoglobin-depleted erythrocyte ghosts (EGs).

To form nano-sized particles, EGs were sequentially extruded through 400 nm and 200 nm polycarbonate porous membranes (VWR Funding, Inc.) 20 times using an Avanti mini extruder (Avanti Polar Lipids, Inc.). The 20 mL of the resulting nano-sized EGs were suspended in a solution comprised of 1 × PBS, 0.1 M Sorenson’s buffer (Na_2_HPO_4_/NaH_2_PO_4_, 140 mOsm, pH ≈ 8), and the FDA approved NIR dye Indocyanine Green (ICG) (Thermo Fisher Scientific Inc., Waltham, MA, USA). The concentration of ICG in this loading buffer was either 20 μM or 1 mM. The solution was centrifuged (≈ 56, 000 × g for 1 hour at 4 °C) again and the supernatant was removed. The pellet was re-suspended in 1 × PBS, centrifuged, and washed one more time before the resulting NETs pellet was re-suspended in 3 mL 1 × PBS, yielding a 10-fold increase in the relative number density of the NETs. Similar to our previous studies, dynamic light scattering was used to estimate the hydrodynamic diameter distribution of NETs suspended in 1 × PBS, and absorption and fluorescence spectroscopy to characterize their optical properties before IV injection into the animals^12–14^.

### Fluorescence stability and biodistribution of NETs

According to our published study^14^, NETs fabricated using the same methods as those reported here did not show any statistically significant changes in time-dependent fluorescence stability after 12 hours of storage at either 4 °C or 37 °C. These findings were in contrast to free ICG, which exhibited about 50% reduction in time-dependent fluorescence stability under the same conditions.

The biodistribution and acute toxicity of NETs on healthy Swiss Webster mice have been previously been examined and reported^15^. In brief, following tail vein injection of free ICG and NETs, animals were euthanized at various time points up to 48 hours. Fluorescence analysis of blood showed that nearly 11% of the injected amount of nano-sized NETs (nNETs) remained in blood at 48 hours post-injection as compared to ≈5% for micron-sized NETs (μNETs). Similarly, at this time point, higher levels of nNETs were present in various organs including the lungs, liver, and spleen. Histological analyses of various organs, extracted at 24 hours post-injection of nNETs and μNETs, did not show any pathological alterations. Serum biochemistry profiles also did not show any elevated levels of biomarkers associated with liver and kidney functions. Values of various hematological profiles remained within the normal ranges following the administration of nNETs and μNETs. Findings from this study suggest that erythrocyte-derived particles can potentially provide a nontoxic platform for delivery of ICG.

### *In Vivo* photoacoustic imaging with Vevo-LAZR/ Vevo-2100

**Figure 1:**
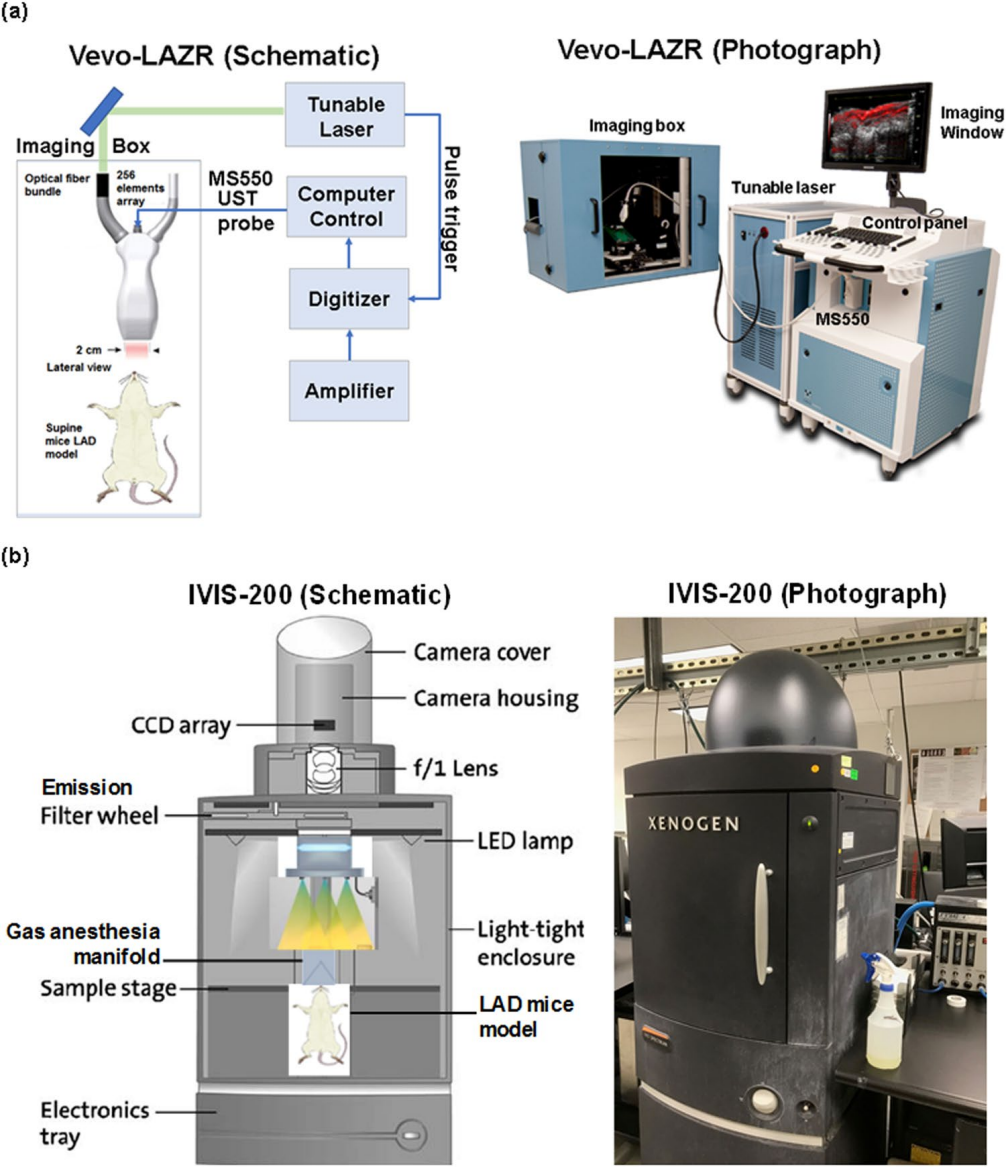
Schematic and photograph of the experimental setup of imaging mice LAD coronary artery with **(a)** Vevo-LAZR and **(b)** IVIS-200.

The Vevo-LAZR LZ550 (FujiFilm VisualSonics Inc.) transducer system was used in this study to generate the photoacoustic images (Fig. 1a). The Vevo-LAZR system is a versatile multi-modality PA imaging platform based on the Vevo-2100 (ultrasound) which combines high resolution anatomical visualization with functional data. The scanning head had array transducers with 256-element and collected 2D PA images with 44-µm axial resolution at 740 frame-per-second (fps). The PA signal was collected up to 6.5 mm from the surface. The data acquisition time was 0.2 seconds. The energy density of the flash-lamp pumped Q-switched Nd-YAG laser at the imaging region of interest was 45 ± 5 mJ for maximum frequency of 20 Hz. This system incorporated photoacoustic imaging into high-resolution ultrasound (US). Photoacoustic images were obtained at 800 nm excitation wavelength with the array transducer of 40 MHz central frequency due to maximal absorption of NETs at this wavelength. Optical parametric oscillator of the LAZR system was pumped by frequency-doubled Nd:YAG laser with a repetition rate of 20 Hz, pulse duration of 4 to 6 ns, spot size of 24 mm^2^. For the PA signal acquisition, the gain was set to 40 dB with 2D gain of 22 dB. The imaging depth of the Vevo-LAZR was set at 10 mm and width of 14.08 mm. A 2D ultrasound imaging was also performed for colocalization of photoacoustic signals.

### *In Vivo* Fluorescence Imaging with IVIS-200

The IVIS-200 system (Xenogen Product of PerkinElmer Inc., Waltham, MA, USA) provides bioluminescence and fluorescence imaging and was used to identify the location of the NETs accumulation within the coronary artery (Fig. 1b). Briefly, *in vivo* left anterior descending (LAD) mouse was placed inside the light-tight imaging chamber of the IVIS-200 system with a field-of-view of 13 cm. Images were taken with a highly sensitive CCD camera system cooled to −90 °C. For fluorescence imaging, the binning factor was set at small (4 × 4 pixels) and peak excitation/emission at 650 nm and 700 nm wavelengths, respectively. Fluorescent images were acquired at exposure times of 0.5, 1, 5, and 10 seconds. An average radiant efficiency was calculated with a unit of (p/sec/cm^2^/sr)/(µW/cm^2^) based on the IVIS-200 images after correction for field flatness.

### Animal preparation prior to imaging

All mice experiments were performed based on an approved protocol by the Administrative Panel on Laboratory Animal Care at the Stanford University School of Medicine (APLAC #18086). Prior to IV injection, radiant efficiency of both 20 µM and 1 mM ICG loaded NETs was measured with IVIS-200 fluorescence imaging system at 650 nm excitation wavelength. These NETs were placed in two separate vials followed by imaging with IVIS-200 at 650 nm wavelength.

Before heart imaging, the chest hair was removed with Nair Hair Removing Cream, and. mice were then anaesthetized by a nose cone delivering 2 L min^−1^ O_2_ gas mixed with 3% isoflurane. Hot water filled balloon was placed on the tail for 2 minutes to dilate the vessel following cleaning the tail with alcohol swap. 150 µL of 20 µM NETs was injected via tail vein using a catheter and a drop of tissue adhesive (3 M Vetbond) to hold the needle in place. The catheter was prepared by taking out the 28 G needle from the syringe and place it into a PVC tube (Luer-Stub Adapters from Sterile Intramedic, Sparks, MD). Only 20 µM NETs was used for fluorescence imaging due to fluorescence quenching from aggregated state of 1 mM ICG in NETs^16^. One hour after injecting the NETs, mice were imaged with VevoSonic/LAZR and IVIS-200 for photoacoustic and fluorescence imaging, respectively. Mice were randomly selected from cages for all experiments. No blinding was performed. All imaging was performed at one hour after the tail vain injection of NETs.

### *In Situ* verification with IVIS-200 and histology

After *in vivo* photoacoustic and fluorescence imaging with the Vevo-LAZR and IVIS-200, mice were sacrificed for further verification. The chest was cut open to image the exposed heart with the IVIS-200 imaging system to proof the *in vivo* fluorescent images. At last the hearts were harvest for histochemical analysis.

### Co-registration with histological analyses

After the experiments, mice heart samples (n = 9) were place in 10% formalin, and submitted for paraffin embedding, sectioning, and staining. Step sections (5 µm thick, n = 16) were collected at different levels beginning at one end of the sample and proceeding to the other end. The sections were mounted on glass microscopic slides, stained with hemotoxylin and eosin (for lipid), trichrome (for collagen), and EVG (for elastin) reagents and covered with Tissue Tek Film as cover slips.

## Results

**Figure 2:**
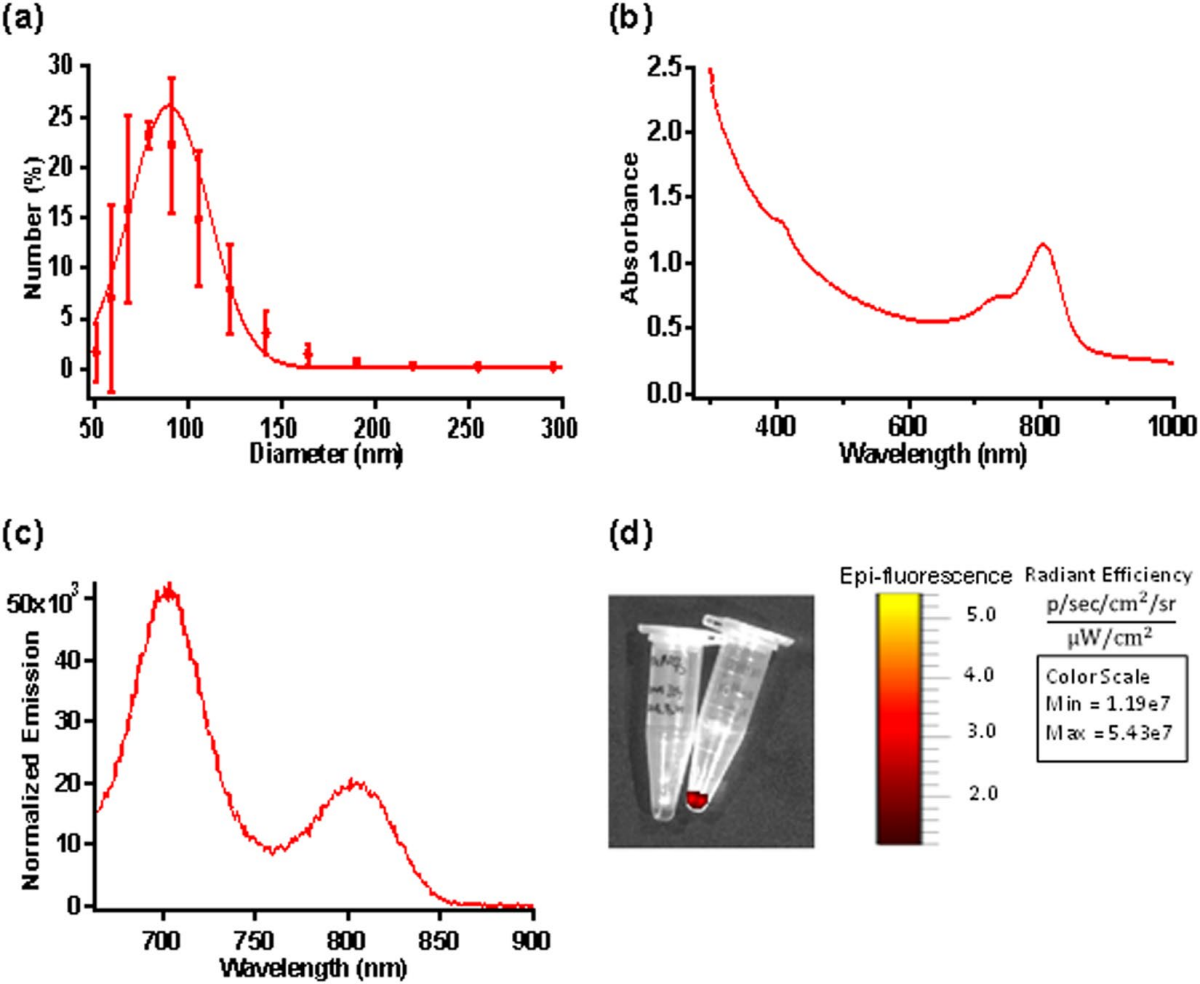
Characterization of NETs. **(a)** Hydrodynamic diameters of NETs measured by dynamic light scattering. We present the averages (circles) with SD values (error bars) associated with three measurements of a sample. A weighted Gaussian distribution was fitted to the measured data (solid trace), indicating a peak diameter of 89.919 ± 3.35 nm. **(b)** Absorption spectra of NETs recorded in 1xPBS. **(c)** Fluorescence emission of NETs in response to photo-excitation at 650 ± 2.5 nm. All measurements were made using NETs that had been fabricated with 20 μM ICG in the loading buffer. **(d)** Before injecting NETs to LAD coronary artery ligation murine model fluorescence intensity of 20 µM (right) and 1 mM (left) NETs were imaged with IVIS-200. However, fluorescence signal was only detected for 20 µM NETs and calculated radiant efficiency of 5.43 × 10^7^ (p/sec/cm^2^/sr)/(µM/cm^2^). Based on this finding 20 µM NETs (150 µL volume) was injected via tail vein to *in vivo* murine models.

NETs have shown peak hydrodynamic diameter distribution of 89.92 ± 3.35 nm based on the dynamic light scattering (Fig. 2a) and strong absorption over 600–800 nm due to the presence of ICG (Fig. 2b). In response to photo-excitation at 650 nm, fluorescence emission peaks at ≈ 700 and 790 nm, corresponding to the respective H-like and monomer forms of ICG were observed^14^ (Fig. 2c). The radiant efficiency was also measured for 20 µM and 1 mM NETs, in two separate vials using IVIS-200 prior to the *in vivo* experiments (Fig. 2d) to identify the correct concentration for NETs dose. We found a radiant efficiency of 5.43 × 10^7^ (p/sec/cm^2^/sr)/(µM/cm^2^) for 20 µM NETs and none for 1 mM NETs, suggesting aggregation-induced quenching for 1 mM NETs. Based on this finding only 20 µM NETs was injected to the mice models.

**Figure 3:**
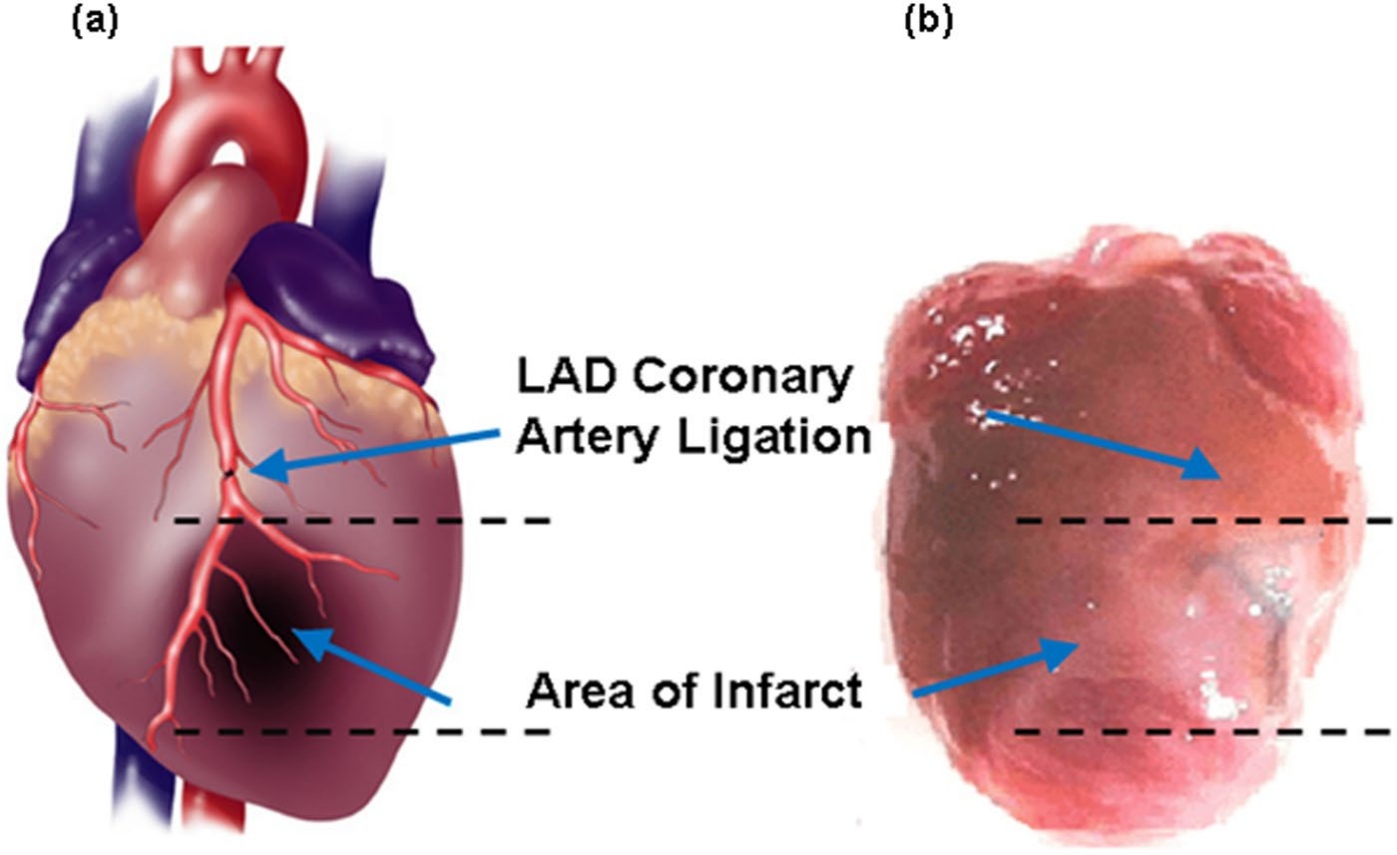
Images of mice heart. **(a)** Schematic diagram (adapted from Wu, Y., Yin, X., Wijaya, C., Huang, M., McConnell, B. K. Acute Myocardial Infarction in Rats. J. Vis. Exp. (48), e2464, doi:10.3791/2464 (2011) for reuse and reprint with permission from Bradley McConnell and JoVE)^30^ and **(b)** photograph (with smart phone) of heart provided the orientation of the ligation location in the LAD coronary artery of the mice heart and the area of infarct (between two dotted lines), respectively.

**Figure 4:**
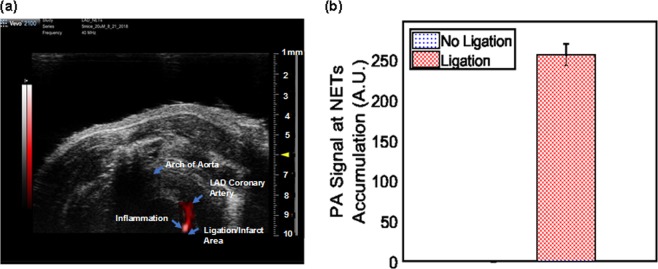
LAD coronary artery mice model was imaged in the chest area with a non-invasive photoacoustic and ultrasound imaging system (VevoSonic 2100/LAZR) at one hour after tail vein injection of NETs. **(a)** A high photoacoustic (PA) signal was detected at the LAD coronary artery above the ligation location (yellow arrow) where NETs appeared to be localized in macrophages, and no PA signal was observed below the ligation or other areas of the heart. **(b)** Statistical data showed 256 × higher PA signal (256 ± 13.73) compared to other locations in the heart.

**Figure 5:**
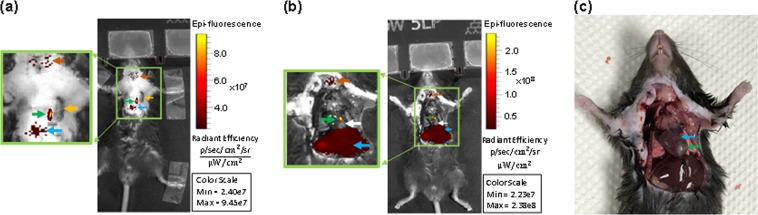
Non-invasive whole-body fluorescence imaging of mice LAD coronary artery ligation model with IVIS-200 at one hour after the tail vein injection of NETs. **(a)** *In vivo* image highlighted NETs were up taken by the inflammatory cells such as macrophages close to ligation location in the coronary artery (green arrow), thymus (orange arrow), liver (blue arrow), and the cut-down location on the chest (yellow arrow) during ligation surgery. **(b)** *In Situ* (the heart is exposed after the chest was cut open) images confirmed our *in vivo* findings. The liver (blue arrow) exhibits traces of NETs uptake compared to the ligation in the heart (green arrow). It also verified that there was no uptake in the infarct region (white arrow). **(c)** Photograph of the heart with a smart phone highlighted the ligation location (blue arrow) in the LAD coronary artery and infarct area with fibrosis (green arrow).

**Figure 6:**
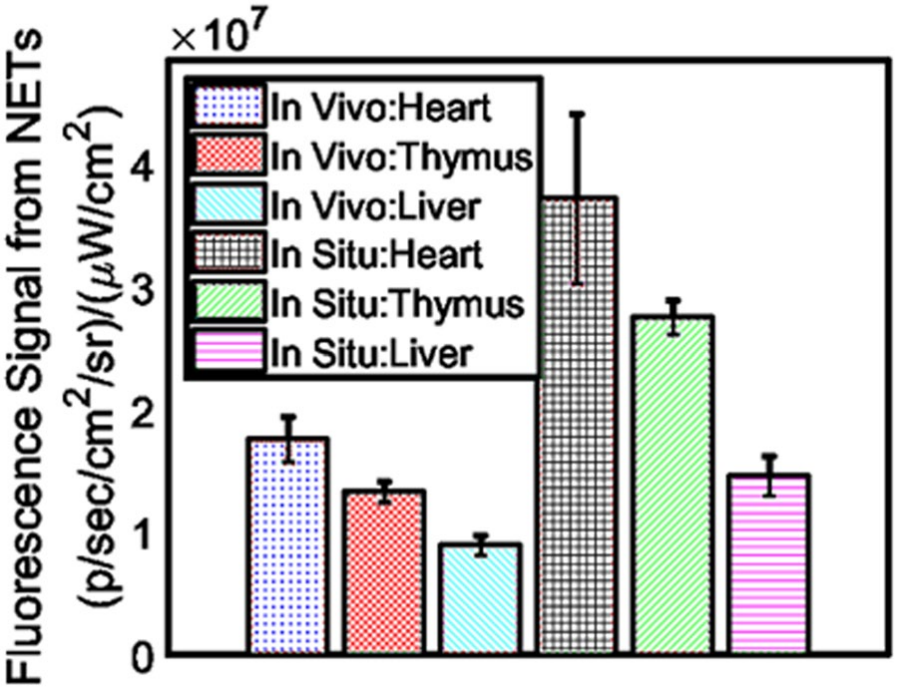
Statistical plot exhibited highest fluorescence signal close to the ligation location in the heart. Liver showed less uptake of NETs compared to thymus. Fluorescence signals from these three organs attained significant level of P-value (P < 0.05).

**Figure 7:**
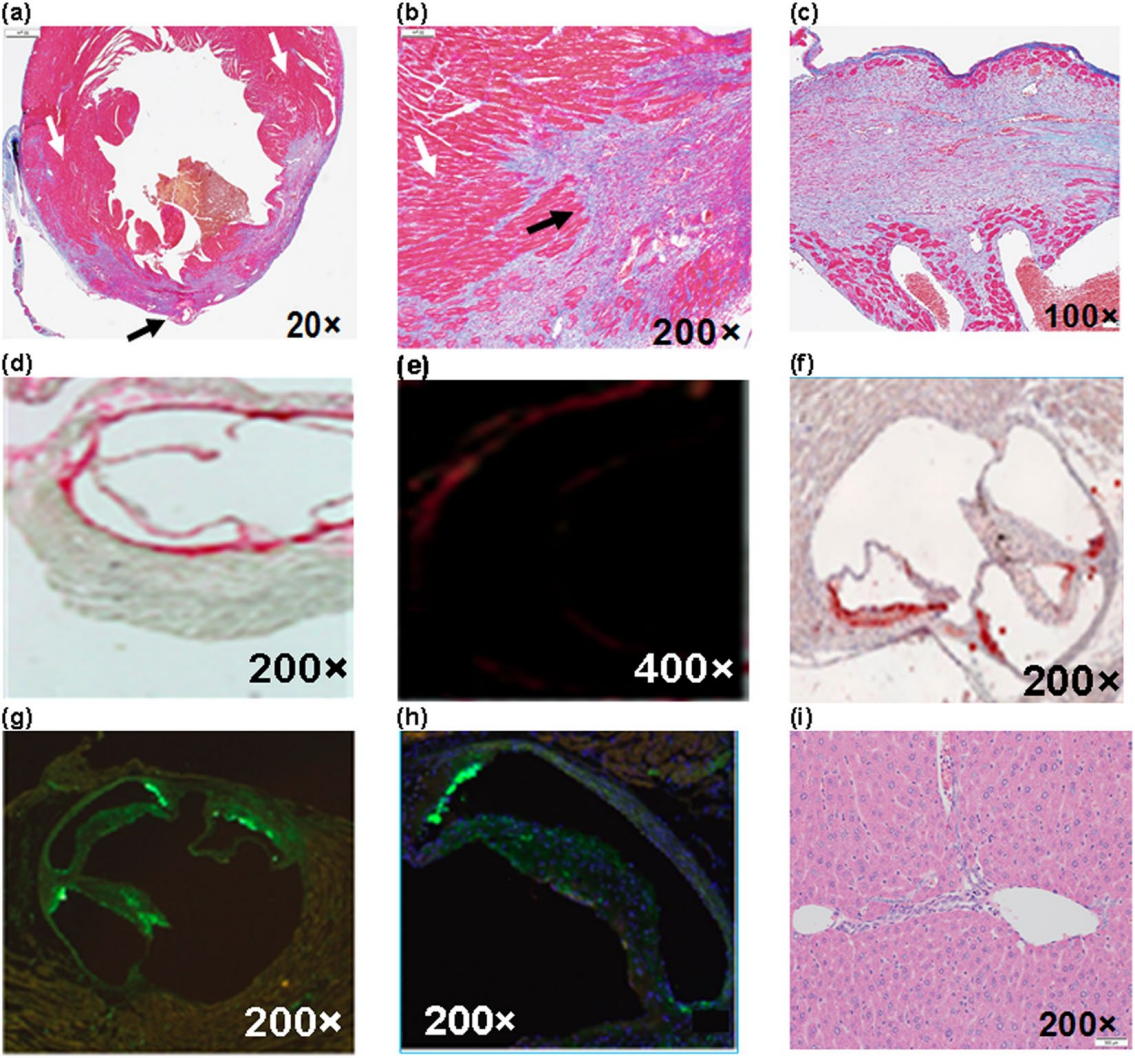
Histochemical analysis. In Trichrome stained heart **(a)** 20×, **(b)** 200× exhibited ligated LAD coronary artery (black arrow) and its surrounding region with infarct stained with blue colored collagen fibers indicative of scarring/tissue necrosis. The viable muscle tissues are highlighted with red stain (white arrow). **(c)** Trichrome stained (100×) septum in between left and right ventricles is highlighted with blue collagen indicative of infract region. **(d)** Picrosirius red indicating degraded collagen (200×) and **(e)** polarized light was used to visualize collagen types (400×). **(f)** Oil Red-O was used to identify lipids in atherosclerotic lesions (200×). Mac-2 staining for **(g)** macrophages present with a dapi and **(h)** macrophage overlay (200×). **(i)** H&E stained (200×) liver histologically demonstrated normal portal tract, surrounding liver parenchyma without necrosis, inflammation, fibrosis or no other pathologic changes. Note: results presented here is based on 20 µM NETs. Note: results presented here is based on 20 µM NETs.

The schematic diagram and photograph of mouse heart illustrated the ligation location at LAD coronary artery with highlighted infarct region (Fig. 3a,b). A 1.5 mm area above the ligation (Fig. 4a, yellow arrow) outlined the accumulation of NETs by exhibiting 256-fold (±13.73) higher (Fig. 4b) photoacoustic signal compared to non-ligated areas. NETs accumulation in the LAD coronary artery, thymus, and liver provided strong fluorescence signals when imaged through intact skin (Fig. 5a). Similar signals were observed in *in situ* mice heart when the chest wall was cut open (Fig. 5b) and were co-registered with *in vivo* images showing a 1.5 mm long area above the ligation of the LAD coronary artery, thymus, and liver. The infarct area with fibrous tissue was identified below the ligation (blue arrow) of the LAD coronary artery (Fig. 5c, green arrow). A 1.6–2 × higher (LAD coronary artery: 3.72 × 10^7^ ± 6.92 × 10^7^ vs. 1.76 × 10^7^ ± 1.85 × 10^6^ (p/sec/cm2/sr)/µW/cm^2^); thymus: 2.75 × 10^7^ ± 1.37 × 10^6^ vs. 1.33 × 10^7^ ± 7.69 × 10^5^ (p/sec/cm2/sr)/µW/cm^2^)) fluorescence signals (Fig. 6) were detected from the *in situ* LAD coronary artery images of the mice heart and thymus compared to *in vivo* images. Decreased signal levels from *in vivo* images are due to attenuated light resulting from scattering by specifically bones from the rib cage, skin, and fat layers. However, liver showed similar fluorescence level (1.46 × 10^7^ ± 1.58 × 10^6^ vs. 8.99 × 10^6^ ± 8.05 × 10^5^ vs. (p/sec/cm2/sr)/µW/cm^2^)) for both *in vivo* and *in situ* images. Statistical data showed that *in vivo* LAD coronary artery of mice heart (1.76 × 10^7^ ± 1.85 × 10^6^) had 1.32-fold (P = 0.006) and 1.96-fold (P = 0.004) higher fluorescence signals compared to thymus (1.33 × 10^7^ ± 7.69 × 10^5^) and liver (8.99 × 10^6^ ± 8.05 × 10^5^), respectively. We considered P < 0.05 as statistically significant for all data analysis. Based on this statistical power, fluorescence signals from all three organs attained significant level of P-value (P < 0.05). Trichrome stained histological images illustrated the infarct region (Fig. 3b, double dotted line) composed of scarring and tissue necrosis at the left ventricle that was immediate below the ligation of the LAD coronary artery (Fig. 7a,b). Intact viable myocytes were only observed in the right ventricle. Trichrome stained septum in between left and right ventricles was highlighted with blue collagen indicative of infarct region with scarring, granulation, and fibrosis (Fig. 7c). Picrosirius red indicating degraded collagen (Fig. 7d) within the affected coronary artery. Polarized light was used to identify degraded collagen type (Fig. 7e). Oli Red-O identified a trace amount of lipids near the ligation area(Fig. 7f). Severe inflammation was detected due to massive macrophage accumulation within the ligation area using Mac-2 and Dapi staining (Fig. 7g,h). H&E stained liver demonstrated normal portal tract (Fig. 7i), with no parenchymal necrosis, inflammation, fibrosis, or other pathologic changes, and hence indicative of the biocompatibility of NETs.

## Discussions

Using combined photoacoustic and fluorescence imaging capabilities could increase the accuracy of detecting occlusions and possibly inflammatory cells such as macrophages. PA signals were generated when exogenous constitute NETs were excited with pulsed laser irradiation causing thermoelastic expansion. The photoacoustic images were were reconstructed similarly to ultrasound images but with a spatial map of optical absorptions by endogenous and exogenous constitutes. The optical absorption and ultrasound scattering of coronary artery tissue makes it optimal for photoacoustic imaging at high resolution and depth (3–5 cm deep)^17–21^, much further than fluorescence imaging which has limited resolution at only a few mm penetration depth^22–24^.

Contrast-enhanced non-invasive photoacoustic imaging is an attractive solution for stenosis detection. One important aspect of using NETs as a contrast agent was that the myocytes did not uptake any of the contrast agent. Therefore, we were able to eliminate high background noise that are usually present using PET tracer such as ^18^F-FDG and opens a new horizon for the whole-body imaging of detection of MI. The accumulation of NETs inside the coronary artery was not from hydrodynamics rather than complete occlusion of the artery leading to inflammation due to macrophage accumulation. This assumption was verified with the findings of the histology study that exhibited macrophages adjacent to the necrosis of myocytes.

Although, we used NETs to detect coronary occlusion including inflammation, this contrast-enhanced photoacoustic imaging can potentially enable information about plaque compositions in addition to structural information based on our previous studies^25,26^. NETs are most likely retained in inflamed tissue through integrin and complement-based adherence to damaged endothelium and/or monocytes, which are themselves attached to the endothelium after injury from the ligation^27^. We were able to image inflammation due to NETs accumulation within coronary arteries with photoacoustic system while most of the circulating NETs have been cleared and allowed us to distinguish symptomatic from asymptomatic coronary arteries.

Our photoacoustic imaging in conjunction with NETs in mice model paved a way for using this technique in larger animals such as rabbits and pigs. In future, we plan to demonstrate that NETs/photoacoustic can provide information on plaque vascularity or inflammation in larger animals, which will lead to translation in to clinical practice for identifying patients with high risk of stroke or transient ischemic attack. Photoacoustic imaging can also be helpful with characterizing cardiac dynamics as well as finding real-time volumetric assessment in MI model and human carotid artery^28,29^. For this to happen, photoacoustic imaging systems will need robust methods of automated signal quantification in three dimensions and reproducibility of the technique.

Nonetheless, we have able demonstrated that NETs in conjunction with photoacoustic imaging showed much higher uptake in the adjacent area to infarct with inflammation compared to liver. Similar results were found through the fluorescence imaging. There were no tissue damages observed in the liver from NETs indicative of biocompatibility. We have demonstrated a potential future for clinical detection of human inflammation from coronary stenosis and offer the possibilities for expanding our toolbox for noninvasive imaging.

## Conclusion

Use of NETs to detect the location of inflammation and stenosis non-invasively may drive a paradigm shift in the diagnosis of CAD, enabling clinical detection and improving risk assessment. The technique described herein will provide invaluable tool enabling researchers to investigate disease processes and clinicians to patient’s risk-stratification.

## Data Availability

The data that are used in this study for analysis are included in this manuscript in the form of Figures and Table.
