# Author Correction: Non-Invasive Photoacoustic Imaging of *In Vivo* Mice with Erythrocyte Derived Optical Nanoparticles to Detect CAD/MI

**DOI:** 10.1038/s41598-020-75966-x

**Published:** 2020-10-30

**Authors:** Yonggang Liu, Taylor Hanley, Hao Chen, Steven R. Long, Sanjiv S. Gambhir, Zhen Cheng, Joseph C. Wu, Georges El Fakhri, Bahman Anvari, Raiyan T. Zaman

**Affiliations:** 1 Department of Medicine, Division of Cardiology, Stanford University School of Medicine, Stanford, CA USA; 2 Department of Bioengineering, University of California, Riverside, CA USA; 3 Department of Radiology, Stanford University School of Medicine, Stanford, CA USA; 4 Department of Pathology, University of California, San Francisco, CA United States; 5 Molecular Imaging Program at Stanford, Stanford University School of Medicine, Stanford, CA USA; 6 Department of Bioengineering, Stanford University School of Medicine, Stanford, CA USA; 7 Stanford Cardiovascular Institute, Stanford University School of Medicine, Stanford, CA USA; 8 Department of Radiology, Harvard Medical School, Boston, MA USA; 9 Gordon Center for Medical Imaging, Massachusetts General Hospital, Boston, MA USA

Correction to: Scientific Reports 10.1038/s41598-020-62868-1, published online 06 April 2020

This Article contains errors in Figure 7: Panels (d), (e), (f), (g) and (h) are incorporated erroneously. Furthermore, the correct panels (b) and (d) are missing in the image. As a result, the Figure legend,

“Histochemical analysis. In Trichrome stained heart **(a)** 20 × , **(b)** 200 × exhibited ligated LAD coronary artery (black arrow) and its surrounding region with infarct stained with blue colored collagen fibers indicative of scarring/tissue necrosis. The viable muscle tissues are highlighted with red stain (white arrow). **(c)** Trichrome stained (100 ×) septum in between left and right ventricles is highlighted with blue collagen indicative of infract region. (d) Picrosirius red indicating degraded collagen (200 ×) and (e) polarized light was used to visualize collagen types (400 ×). **(f)** Oil Red-O was used to identify lipids in atherosclerotic lesions (200 ×). Mac-2 staining for **(g)** macrophages present with a dapi and **(h)** macrophage overlay (200 ×). **(i)** H&E stained (200 ×) liver histologically demonstrated normal portal tract, surrounding liver parenchyma without necrosis, inflammation, fibrosis or no other pathologic changes. Note: results presented here is based on 20 µM NETs. Note: results presented here is based on 20 µM NETs.”

should read:

“After the photoacoustic and fluorescence imaging the heart was harvested for histiochemical analysis. Trichrome stained heart **(a)** 20 × , **(b)** 100 × , **(c)** 200 × exhibited ligated LAD coronary artery (green arrow) and its surrounding region of infarct area (black arrow) stained with blue colored collagen fibers indicative of scarring/infarction. The viable muscle tissues are highlighted with red stain (white arrow). **(d)** Trichrome stain of the heart (20x), showing left ventricle (red arrow) with blue staining collagen fibers indicative of scarring/infarction and right ventricle (blue arrow) with bright red staining of intact viable myocytes (muscle tissue). Early thrombus (yellow arrow) is also observed. **(e)** Trichrome stained (100x) septum in between left and right ventricles is highlighted with blue collagen indicative of infract region. **(f)** H&E stained (200x) liver histologically demonstrating normal portal tract, surrounding liver parenchyma without necrosis, inflammation, fibrosis or no other cell necrosis.”

The correct version of Figure 7 and the correct Figure legend appear below as Figure 1.

**Figure 1:**
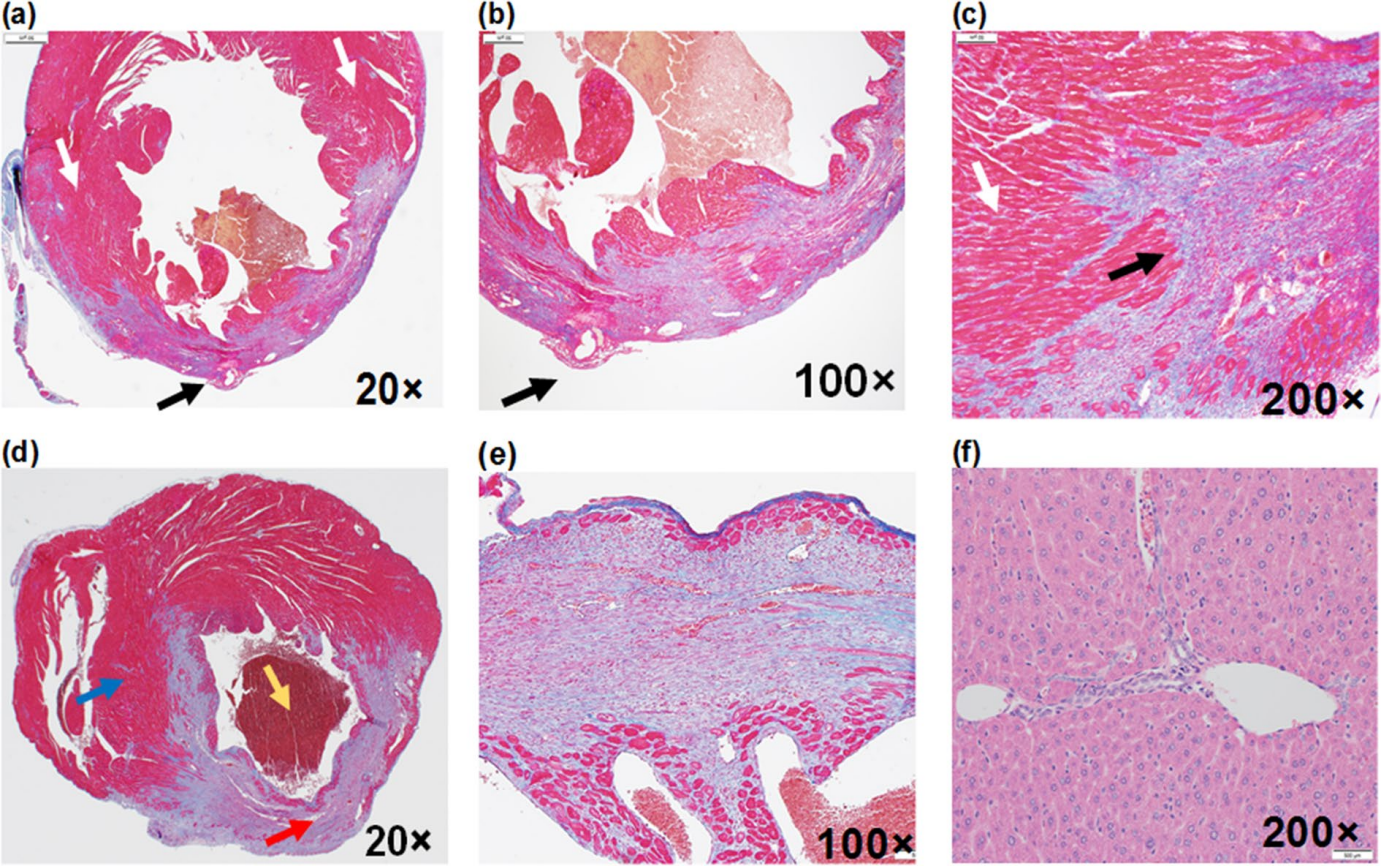
After the photoacoustic and fluorescence imaging the heart was harvested for histiochemical analysis. Trichrome stained heart (**a**) 20×, (**b**) 100×, (**c**) 200× exhibited ligated LAD coronary artery (green arrow) and its surrounding region of infarct area (black arrow) stained with blue colored collagen fibers indicative of scarring/infarction. The viable muscle tissues are highlighted with red stain (white arrow). (**d**) Trichrome stain of the heart (20×), showing left ventricle (red arrow) with blue staining collagen fibers indicative of scarring/infarction and right ventricle (blue arrow) with bright red staining of intact viable myocytes (muscle tissue). Early thrombus (yellow arrow) is also observed. (**e**) Trichrome stained (100×) septum in between left and right ventricles is highlighted with blue collagen indicative of infract region. (**f**) H&E stained (200×) liver histologically demonstrating normal portal tract, surrounding liver parenchyma without necrosis, inflammation, fibrosis or no other cell necrosis.

